# Skin cancer classification based on an optimized convolutional neural network and multicriteria decision-making

**DOI:** 10.1038/s41598-024-67424-9

**Published:** 2024-07-27

**Authors:** Neven Saleh, Mohammed A. Hassan, Ahmed M. Salaheldin

**Affiliations:** 1 Systems and Biomedical Engineering Department, Higher Institute of Engineering, EL Shorouk Academy, Cairo, Egypt; 2 Electrical Communication and Electronic Systems Engineering Department, Engineering Faculty, October University for Modern Sciences and Arts, Giza, Egypt; 3 Biomedical Engineering Department, Faculty of Engineering, Helwan University, Cairo, Egypt

**Keywords:** Skin cancer, CNN, Grey wolf optimizer, Machine learning, Multicriteria decision-making, Skin cancer, Cancer, Diseases, Health care, Engineering

## Abstract

Skin cancer is a type of cancer disease in which abnormal alterations in skin characteristics can be detected. It can be treated if it is detected early. Many artificial intelligence-based models have been developed for skin cancer detection and classification. Considering the development of numerous models according to various scenarios and selecting the optimum model was rarely considered in previous works. This study aimed to develop various models for skin cancer classification and select the optimum model. Convolutional neural networks (CNNs) in the form of AlexNet, Inception V3, MobileNet V2, and ResNet 50 were used for feature extraction. Feature reduction was carried out using two algorithms of the grey wolf optimizer (GWO) in addition to using the original features. Skin cancer images were classified into four classes based on six machine learning (ML) classifiers. As a result, 51 models were developed with different combinations of CNN algorithms, without GWO algorithms, with two GWO algorithms, and with six ML classifiers. To select the optimum model with the best results, the multicriteria decision-making approach was utilized to rank the alternatives by perimeter similarity (RAPS). Model training and testing were conducted using the International Skin Imaging Collaboration (ISIC) 2017 dataset. Based on nine evaluation metrics and according to the RAPS method, the AlexNet algorithm with a classical GWO yielded the optimum model, achieving a classification accuracy of 94.5%. This work presents the first study on benchmarking skin cancer classification with many models. Feature reduction not only reduces the time spent on training but also improves classification accuracy. The RAPS method has proven its robustness in the problem of selecting the best model for skin cancer classification.

## Introduction

A frequent type of cancer that is often caused by sun exposure is skin cancer^1^. Melanoma and nonmelanoma cases are classified according to the clinical category^1–3^. Even though melanoma is less common than nonmelanoma, it is the deadliest^2–5^. In 2019, the average death rate was 4740 males and 2490 females because of melanoma^1^. Indeed, the high cure rate of skin cancer mainly depends on early detection. In fact, dermatologists usually depend on dermoscopic images for melanoma detection and diagnosis. Despite this being a common method for diagnosis, it is not accurate. This is due to the high similarity of features between melanoma and nonmelanoma lesions. Additionally, image noise in the form of blood vessels and hair^4^ can lead to confusion. The accuracy of correctly diagnosing melanoma using visual examination is less than 80%^6^.

To improve the diagnosis of melanoma, several automated systems have been proposed for identifying skin lesions to aid dermatologists. In this context, machine learning (ML) and deep learning methods were adopted. Various publications have classified melanoma and nonmelanoma lesions using ML methods. However, many limitations arise in terms of shallow training performance, extensive image processing, and variations in interclass and intraclass dermoscopic images^7^. Relevant studies were conducted based on ML methods. For instance, the K-nearest neighbor (K-NN) method has been employed for skin cancer classification, and it has achieved an accuracy of 98%^8^.

Unlike in ML algorithms, large sets of raw data are used as inputs, and end-to-end training and automatic feature learning are the main advantages of deep learning algorithms^9^. In fact, the CNN technique has extensive applications in disease diagnosis. The performance of CNNs in a variety of cancer detection and classification tasks, such as breast cancer^10^, prostate cancer^11^, liver lesions^12^, and lung cancer^13^, is particularly encouraging. According to Sudharshan et al.^14^, employing CNN models enhances the performance of diagnostic systems. The general CNN framework consists of layers for feature extraction, feature mapping, and subsampling^15^. However, feature extraction and mapping play a significant role in the outcome of the network. Taking this into account, the performance of some algorithms is influenced by the number of features, and rabbits in turn require feature reduction. The benefits of feature reduction include data storage space reduction, computation time reduction, removing noisy data, and improving the classification process^16^.

Many techniques, including filtering, wrapping, and embedding, have been proposed for feature reduction. The heuristic search algorithm is a wrapper technique^16^. Metaheuristic methods, or stochastic methods, are adopted for tackling various problems as solvers when optimization is needed. These methods do not rely on the mathematical characteristics of the object^17^. Swarm intelligence-based stochastic approaches are the most widely used. The primary evolutionary origins of swarm-based approaches are drawn from animal foraging, hunting, and survival of the fittest^18^. Recent examples of well-known methods include the Harris hawk optimizer (HHO)^17^, the whale optimization algorithm (WOA)^19^, ant colony optimization (ACO)^20^, fruit fly optimization (FFO)^21^, and the grey wolf optimizer (GWO)^22^.

This study was conducted to classify skin cancer using different types of CNNs. The main hypothesis of the study is whether optimizing the features will enhance the performance of the CNNs. The other question that arises is which is the best paradigm for classifying skin cancer. To answer these questions, we must select the best model according to the metrics of evaluation performance. In this context, the multicriteria decision-making (MCDM) approach is appropriate for selection. Therefore, the aim of this study was to select the best paradigm for skin cancer classification by optimizing the features and employing the MCDM approach. Thus, the authors employed three main approaches in this study: deep learning and ML, metaheuristic optimization, and MCDM. For deep learning, the AlexNet^23^, Inception V3^9^, MobileNet V2^24^, and ResNet 50^2^ architectures were proposed. In addition, distinctive types of ML were employed. Among the available optimizers, the grey wolf optimizer was proposed^22^. For the MCDM stage, a recent method called ranking the alternatives by perimeter similarity (RAPS)^25^ was investigated.

The main contributions of this study are summarized as follows: (1) different networks of CNNs with different combinations of hyperparameters are used to minimize biasing errors; (2) features are optimized using a recent optimizer with different scenarios to improve classification accuracy; (3) multiple ML classifiers with various configurations are used for image classification; (4) for the first time, a significant number of models for the classification of skin cancer have been constructed in one study using different configurations; (5) a recent method of the MCDM approach was used to select the best CNN model; (6) the importance of feature reduction in the classification of skin cancer and selection of the best model has been highlighted; and (7) the automated diagnosis of skin cancer has improved, which in turn impacts diagnosis and patient healing.

The article is organized as follows: Related works on the problem at hand are presented in Related Work. The background of the utilized methods and tools is covered in Background. The methodology, which includes different approaches, is introduced in detail in Materials and Methods. The experiments and results of each utilized approach associated with the discussion of the obtained results are demonstrated in Results and Discussion. Finally, the study conclusions and future work are given in Conclusion.

## Related work

Various studies have been presented on the problem of skin cancer detection and classification. Additionally, different CNN models were tested for this problem because most studies concluded that there was a significant enhancement in the classification accuracy of CNNs^26^. In 2020, Zhang et al.^27^ presented a new methodology based on a CNN for detecting skin cancer. The method combines the WOA with a CNN to optimize its usage. Through integration, errors are minimized due to the optimum selection of weights and biases. The results demonstrated the efficiency of the study. Singh et al.^18^ developed a model to classify melanoma using inception and residual networks. The authors have added 40 layers for the classifiers. The models were tested using a dataset from the International Skin Imaging Collaboration (ISIC) for the years 2018, 2019, and 2020. The results demonstrated the effectiveness of the developed models compared to other algorithms. The performance of the models was evaluated through accuracy, specificity, sensitivity, and intersection over union (IOU) metrics.

In 2023, a set of dermoscopic images was split into melanoma and nonmelanoma images^2^. Two feature extraction and fine-tuning strategies have been used in transfer learning-based techniques. The classifiers EfficientNet B6, ResNet 50, DesNet 121, and Inception-ResNet V2 were tested for both techniques. The classifiers were evaluated through area under the curve (AUC) and receiver operating characteristic (ROC) curve analyses using datasets from ISIC 2019 and 2020. The results proved the robustness of EfficientNet B6 for both feature extractions, with an AUC of 0.9174, and fine tuning, with an AUC of 0.9681. In another relevant study, Dong et al.^3^ proposed modifications to Inception-ResNet-V1 combined with quantum computing to improve the performance of CNNs. The support vector machine (SVM) method was employed in place of the network’s fully connected (FC) layer. Additionally, to lessen the effects of data imbalance, the authors used weighted sampling and data augmentation techniques. This study adopted three experiments for the ISIC 2019 dataset to test the proposed approach. The best performance was measured for the experiment using a processed dataset, for which the accuracy was 98.76%. An improved artificial rabbit optimizer was introduced to optimize features for detecting skin cancer. The MobileNetV3 network has been employed to detect melanoma-related skin cancer. Three datasets were tested to prove the model’s validity. The model yielded rational results with accuracies of 87.17%, 96.79%, and 88.71% for the different datasets^4^.

In another relevant work, a stacked convolutional neural network was proposed for detecting melanoma^5^. First, the authors used pretrained CNN models to demonstrate the performance of the modified CNN model. Second, the stacked CNN, which uses 2D layers, was presented. The improved network was evaluated for two datasets, MINST HAM1000 and ISIC 2020. A t test was carried out to determine the significance of the proposed network compared to traditional networks. A new framework for skin cancer detection was presented using MobileNetV3 with a novel algorithm for feature selection^28^. Once the features have been extracted, a modified Hunger Games Search (HGS) based on Particle Swarm Optimization (PSO) and Dynamic-Opposite Learning (DOLHGS) was utilized as the input. The proposed paradigm was tested on the ISIC 2016 and PH2 datasets. Multiple classification models based on deep learning were presented in^29^. In this study, a novel CNN was presented for classifying skin cancer. The network is called a deep learning-based skin cancer classification network (DSCC_Net). The network was compared to six well-known CNN models: ResNet-152, VGG-19, VGG-16, EfficientNet-B0, MobileNet, and Inception-V3. DSCC_Net was tested using three different datasets (ISIC 2020, DermIS, and HAM10000). The network has yielded good performance compared to the other networks.

Another study classified skin cancer based on training various deep learning-based methods. Two datasets were used for validation: ISIC 2019 and ISIC 2020^30^. In 2022, Javed Rashid et al.^24^ published a study on detecting skin cancer using a transfer learning technique. The authors proposed a novel transfer learning CNN using MobileNet V2. The ISIC 2020 dataset was used to test the model. Classification of different types of skin cancer was introduced by Naeem et al.^31^, in which a novel CNN called SCDNet was proposed based on VGG16. Different CNN models, such as ResNet50 and VGG19, were compared against the developed model. The dataset from ISIC 2019 was used for comparison and validation of the network. Thus, CNNs have been employed in different versions for skin cancer detection. This has encouraged us to employ various CNN models. The findings of feature selection optimization are also promising. Additionally, we noted that the MCDM approach was not adopted for selecting the best model out of the various models. In this way, the authors decided to combine these approaches to develop an improved automated system for skin cancer classification.

## Background

This work adopted three approaches for implementation. First, a deep learning approach is used to construct CNNs with different structures. Second, an optimization is employed for feature reduction and selection. Third, MCDM was used to select the best paradigm for classifying skin cancer. A general overview of each approach is given in the following subsections. The ML classifiers utilized in the classification process were introduced in the Materials and Methods section.

### Convolution Neural Network (CNN)

The CNN is the most popular deep learning algorithm. The origin of the network is referred to as the appearance of LeNet in 1989^32,33^. The main advantage of CNNs is their ability to extract features automatically without guiding supervision. In addition, compared to other neural networks, CNNs are significantly easier to construct on a large scale^32^. In application, CNNs have widespread applications in various fields, such as disease detection and classification^2,9–11,34^, transportation^35^, facial recognition^36^, and speech recognition^37^. A common architecture structure of CNNs is convolutional layers followed by subsampling (pooling) layers followed by FC layers^32^. Many modified CNN layouts have been created to improve the performance of CNNs. Examples of modified CNNs include AlexNet, ResNet 50, Inception V3/V4, GoogleNet, MobileNet-v2, VGG 16/19, SqueezeNet, EfficientNet-B0, and HRNetV2^9,29,32^. The study employs AlexNet^23^, Inception V3^9^, MobileNet V2^24^, and ResNet 50^38^. Therefore, a brief description of each network is given in the following subsections.

#### AlexNet

AlexNet was created by Krizhevesky et al.^39^ in 2012. To enhance the learning capability, many parameters have been optimized in addition to the increase in length. The depth of the AlexNet is 8 layers, and the basic input image size is 227 × 227 × 3. Several origin-related drawbacks of AlexNet include the extension of the five phases of feature extraction to seven stages^32^. In AlexNet, ReLU is used as a nonsaturating activation function. Moreover, large filters 5 × 5 and 11 × 11 are employed by the AlexNet^32,40^.

#### Inception-v3

Inception V3 was designed depending upon its previous version, Inception V1/2. Improvements include reducing the computational cost by selecting small filter sizes of 1 × 5 and 1 × 7. In addition, the proposed method utilizes a small convolution layer (1 × 1) before large filters are used. As a result, compared with that of Inception V2, the performance of the model is reduced by 1.38 times^41^. However, the network comprises approximately 20 million parameters^42^. The soft-max layer is used for classification, and batch normalization is used for the activation layer^42^. Generally, the inception V3 is composed of 48 layers with an image size of 229 × 229 × 3^9,32^.

#### ResNet 50

The residual neural network (ResNet 50) was introduced by He et al.^26^ in 2016. The idea behind this network is to improve the performance by adding residual connections between layers. As a result, losses decrease, knowledge gain increases, training performance improves, and the model becomes robust against overfitting^42^. Several layers compose the ResNet model. It starts with 34 layers and ends with 1202 layers. This variation leads to changes in the residual block number and basic operations. The most common type is ResNet 50, which is composed of 49 convolutional layers and one FC layer^43^.

#### MobileNet V2

One form of CNN that works for optimizing mobile devices is MobileNet V2. The main advantage of this network is its ability to classify images with less computational effort^44^. In this way, a supercomputer is not mandatory. The architecture of the network comprises depthwise and pointwise convolution layers^45^. The network was developed based on MobileNet V1. However, MobileNet V2 is more reliable than MobileNet V1. This is due to two factors: linear bottlenecks between the layers and shortcut connections between the bottlenecks^44,45^.

### Grey wolf optimizer

Swarm intelligence (SI) is a powerful form of computational intelligence inspired by the behavior of a natural swarm^22^. The main idea of SI-based algorithms is to simulate the behavior of swarm members when finding food or hunting prey^46^. This principle is applied by numerous algorithms to optimize a solution to a problem. Examples of SI algorithms include the ant colony algorithm (ACO)^47^, the WOA^27^, the artificial bee colony (ABC)^48^, and the grey wolf optimizer (GWO)^22,49^. In 2014, Mirjalili et al.^50^ introduced the GWO, a new SI-based algorithm. In nature, wolves are hunting prey optimally. The algorithm applies this principle by organizing wolves’ roles in the pack according to the hierarchy^50,51^. Based on the many roles that wolves play in the hunting process, the members of the pack in GWO are separated into four groups. The distinctive groups are alpha, beta, delta, and omega, with alpha denoting the greatest hunting solution^49–51^. The wolf’s category beta assists in hunting decisions, while the third-class delta surrounds the first and second classes for scouting and hunting. Finally, the omega wolves play the lowest role by protecting the backs of the previous wolves. Thus, the roles of wolves in hunting prey can be identified as tracking, pursuing, and assaulting^49^. The mathematical modeling of the GWO according to the social behavior of the wolves is explained in the following steps^22,49–51^.

Assume that the initial location of a wolf to a prey in a circle is provided by the distance ***D,*** as shown in Eq. (1). Due to the chasing process, the location of the wolf is updated through Eq. (2).

1 $$ D = \left| {C \cdot X_{P} \left( t \right) - X\left( t \right) } \right| $$

2 $$ X\left( {t + 1} \right) = X_{P} \left( t \right) - A \cdot D $$

where ***D*** is the distance between the wolf and the prey, ***t*** is the number of iterations, ***X(t)*** is the current location of the wolf, ***Xp(t)*** is the location of the prey, and ***X(t* + *1)*** is the next location of the wolf. The coefficients ***A*** and ***C*** are calculated by Eqs. (3) and (4), respectively.

3 $$ A = \left( {2 \times a \times r_{1} } \right){-}a $$

4 $$ C = 2 \times r_{2} $$

To improve the movement of the wolf, vectors ***r***_***1***_ and ***r***_***2***_ are randomly selected between 0 and 1. The coefficient ***a*** provides a linear decay range from 2 to 0, as presented in Eq. (5), where ***t*** is the number of iterations and ***T*** is the maximum number of iterations.

5 $$ a = 2{-}\left( \frac{2t}{T} \right) $$

According to the previous equations and with numerous iterations, the locations of all wolves (alpha, beta, delta, and omega) are determined and updated toward the prey’s location using Eq. (6).

6 $$ \left. {\begin{array}{*{20}c} {D_{\alpha } = \left| {C_{1} X_{\alpha } - X} \right|} \\ {D_{\beta } = \left| {C_{2} X_{\beta } - X} \right|} \\ {D_{\delta } = \left| {C_{3} X_{\delta } - X} \right|} \\ \end{array} } \right\} $$

7 $$ \left. {\begin{array}{*{20}c} {X_{1} = \left| {X_{\alpha } - A_{1} \cdot D_{\alpha } } \right|} \\ {X_{2} = \left| {X_{\beta } - A_{1} \cdot D_{\beta } } \right|} \\ {X_{3} = \left| {X_{\Omega } - A_{1} \cdot D_{\Omega } } \right|} \\ \end{array} } \right\} $$

where a wolf alpha and a wolf omega are separated by ***D***_***α***_, a wolf beta and a wolf omega are separated by a distance ***D***_***β***_, and a wolf delta and a wolf omega are separated by a distance ${\mathbf{D}}_{{{\varvec{\updelta}}}}$. The new optimum locations for the alpha, beta, and delta wolves are defined as ***X***_***1***_, ***X***_***2***_, and ***X***_***3, respectively,***_ as illustrated in Eq. (7). By assuming that the best solutions for each category are found, other wolf locations are determined, as shown in Eq. (8).

8 $$ X\left( {t + 1} \right) = \left( {X_{1} + \, X_{2} + \, X_{3} } \right)/3 $$

### Multi-criteria decision-making approach

The problem of selecting an alternative among numerous options requires a comprehensive solution. To resolve this problem, many aspects should be considered, including the criteria of selection and their weights. The MCDM approach tackles such problems with many techniques to promote no biasing decision-making. The beginning of the MCDM was in the early eighteenth century^25^. Modern common techniques were first introduced in 1965 by developing elimination and choice in expressing reality (ELECTRE). Several modifications were added to ELECTRE I to produce ELECTRE II, ELECTRE III, ELECTRE IV, and ELECTRE as separate models^25^. In 1968, MacCrimmon created a different technique known as simple additive weighting (SAW)^52^. In the 1970s, Saaty developed the analytic hierarchy process (AHP) as a tool for MCDM^53,54^. Since then, numerous methods have been introduced, including but not limited to the technique for order preference by similarity to the ideal solution (TOPSIS), multi objective optimization by ratio analysis (MOORA), the preference ranking organization method for enrichment evaluation (PROMETHEE), data envelopment analysis (DEA), case-based reasoning (CBR), and the additive ratio assessment system (ARAS)^53,54^.

As the degree to which a criterion is preferred obviously affects the decision-making process, assessing the weight of the criterion is an essential stage in the MCDM. Objective-based methods are widely used for criteria weighting. The entropy method and criterion importance via intercriteria correlation (CRITIC) are common examples of this method^53^. To implement a standard MCDM method, one must first identify the problem, then create acceptable criteria, weigh the criteria, identify potential solutions, and use mathematical formulas to apply a chosen MCDM technique^55^. One of the most recent MCDM techniques is called ranking alternatives by perimeter similarity (RAPS)^25^. In 2021, Urošević et al.^56^ developed the RAPS method for its first application in the mining industry. The application of the RAPS method is explained in the following steps^25,55,56^:

**Step 1**: Normalize the initial data that describe the alternatives against the criteria. Considering that the selected criteria are either beneficial, i.e., maximization is needed, or nonbeneficial, i.e., minimization is required^53^. Equations (9) and (10) present normalizations for both beneficial and nonbeneficial criteria, respectively.

9 $$ r_{ij} = x_{ij} /Max_{i} \left( {x_{ij} } \right),\quad i = 1, \ldots .,n \& \, j = 1, \ldots ..,m\quad \left( {{\text{beneficial}}} \right) $$

10 $$ r_{ij} = Min_{i} \left( {x_{ij} } \right)_{{}} /x_{{i{\text{j}}}} \quad i = 1, \ldots .,n \& \, j = 1, \ldots ..,m\quad \left( {{\text{nonbeneficial}}} \right) $$

where ***r*** represents the normalized value of input ***x*** for an alternative ***i*** for ***n*** alternatives against a criterion ***j*** for ***m*** criteria.

**Step 2**: Calculate a weighted normalized matrix ***u***_***ij,***_ where ***w***_***j***_ represents each criterion weight, as shown in Eq. (11).

11 $$ u_{ij} = w_{j} \times \, r_{ij} $$

**Step 3**: Eligate each element of the best solution (q_j_) using Eq. (12), which in turn produces an optimal solution (Q) through Eq. (13).

12 $$ q_{j} = max \, u_{ij} , \quad i = 1, \ldots ...,n \& j = 1, \ldots .., \, m $$

13 $$ Q = \left\{ {q_{1} , \, q_{2} , \, q_{3} , \ldots ., \, q_{m} } \right\} $$

**Step 4**: Decomposition of an optimal alternative into two subcomponents ***Q***^***max***^ and ***Q***^***min***^ is performed. The maximum number of beneficial criteria is denoted by ***k*** to produce ***Q***^***max***^; meanwhile, the maximum number of nonbeneficial criteria is denoted by ***h* = *m–k*** to yield ***Q***^***min***^. The vector Q is the union of the two subcomponents, as indicated by Eq. (14).

14 $$ Q = Q^{max} \cup Q^{min} = \left\{ {q_{1} , \, q_{2} , \ldots \ldots .,q_{k} } \right\} \cup \left\{ {q_{1} , \, q_{2} , \ldots \ldots ,q_{h} } \right\}; \, k + h = m $$

**Step 5**: As in the previous step, each alternative is broken down into subsets ***U***^***max***^ and ***U***^***min***^ to produce ***U*** values, as shown in Eq. (15).

15 $$ U_{i} = U_{i}^{max} \cup U_{i}^{min} = \left\{ {u_{i1} , \, u_{i2} , \ldots ..,u_{ik} } \right\} \cup \left\{ {u_{i1} , \, u_{i2} , \ldots .,u_{ih} } \right\}; \, i = 1, \ldots .,n $$

**Step 6:** This step pertains to the component's magnitude and requires the calculation of every element that makes up the best option, as shown in Eqs. (16) and (17). The magnitude of each component is determined using Eqs. (18) and (19).

16 $$ Q_{k} = \sqrt {q_{1}^{2} + q_{2}^{2} + \cdots + q_{k}^{2} } $$

17 $$ Q_{h} = \sqrt {q_{1}^{2} + q_{ 2}^{2} + \cdots + q_{h}^{2} } $$

18 $$ Ui_{k} = \sqrt {u^{2}_{i1} + u_{i2}^{2} + \cdots + u^{2}_{ik} } ;\quad i = 1, \ldots \ldots ,n $$

19 $$ U_{ih} = \sqrt {u_{i1}^{2} + u_{i2}^{2} + \cdots + u^{2}_{ih} } ;\quad i = 1, \ldots ..,n $$

**Step 7**: Final sorting of alternatives is achieved by applying Eqs. (20)–(22). In terms of alternatives, the right-angle triangle perimeter ***P*** is the best option. Equation (20) gives the expression for components ***Q***_***k***_ and ***Q***_***h***_, which correspond to the base and perpendicular sides of this triangle, respectively. For each alternative, Eq. (21) is used to calculate an alternative perimeter, ***P***_***i***_. The ratio between ***P***_***i***_ and ***P*** is used to determine an alternative ranking index ***PS***_***i***_***,*** as demonstrated in Eq. (22).

20 $$ P = Q_{k} + Q_{h} + \sqrt {Q_{k}^{2} + Q_{h}^{2} } $$

21 $$ Pi = Ui_{k} + U_{ih} + \sqrt {U_{ik}^{2} + U_{ih}^{2} } $$

22 $$ PS_{i} = P/P_{i} ;\quad i = 1, \ldots ..,n $$

## Materials and methods

**Figure 1:**
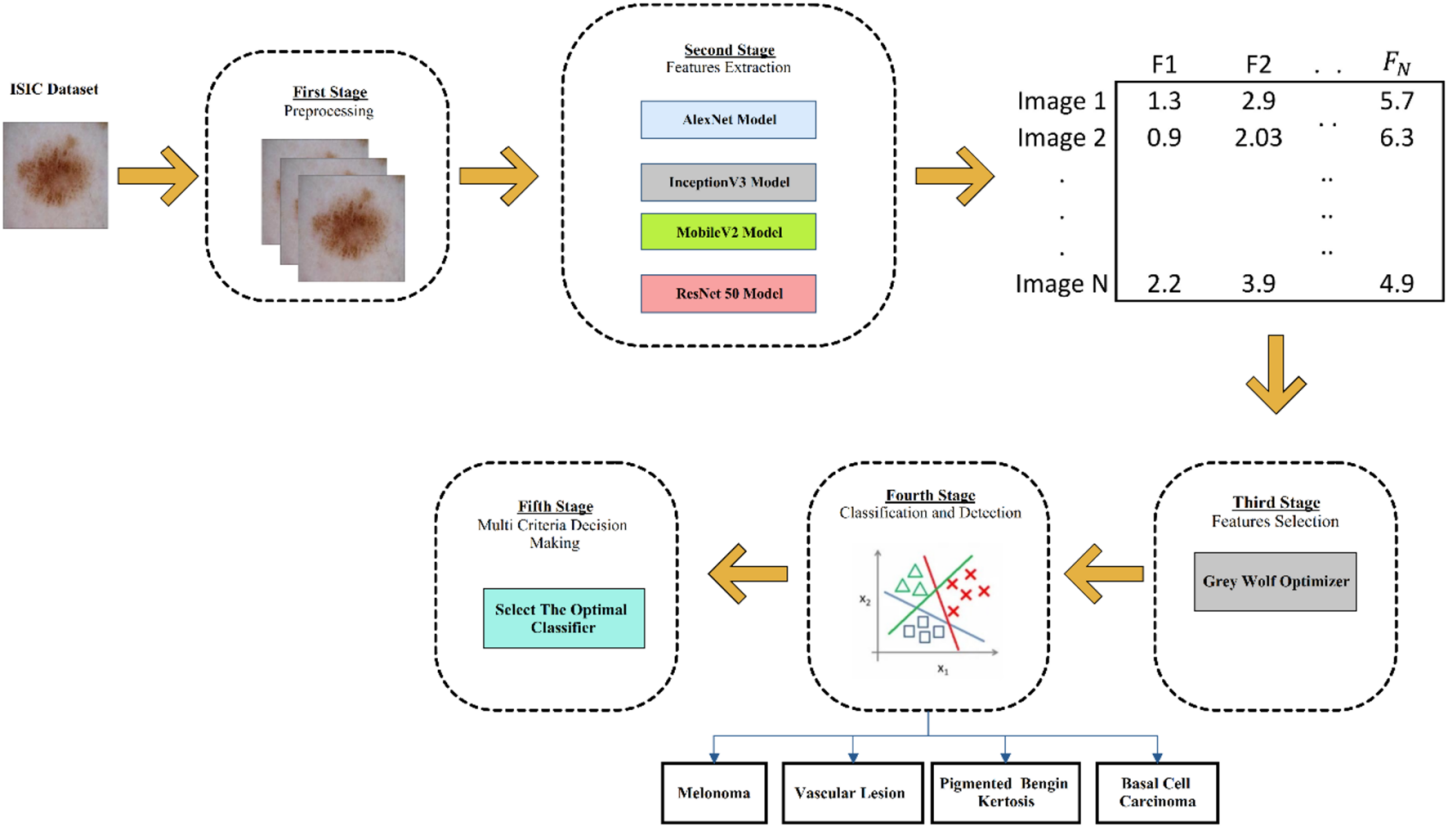
The proposed methodology for skin cancer classification in five stages, including distinctive methods.

This study presents a framework for classifying skin cancer. Various approaches have been applied to the present work. Four algorithms of the CNN, the WGO for feature optimization, different ML classifiers, and the MCDM for selecting the optimum model. An overview of the proposed methodology is given in Fig. 1. As noted, the methodology was conducted in five stages, including distinctive methods. The first stage involved data processing; the second stage involved feature extraction via the CNN algorithms; the third stage involved feature selection via the application of GWO; and the fourth stage involved classifying the dermoscopic images into four classes via different ML classifiers. The last stage involved selecting the optimum model among all the developed models via applying the MCDM.

### Dataset description

**Figure 2:**
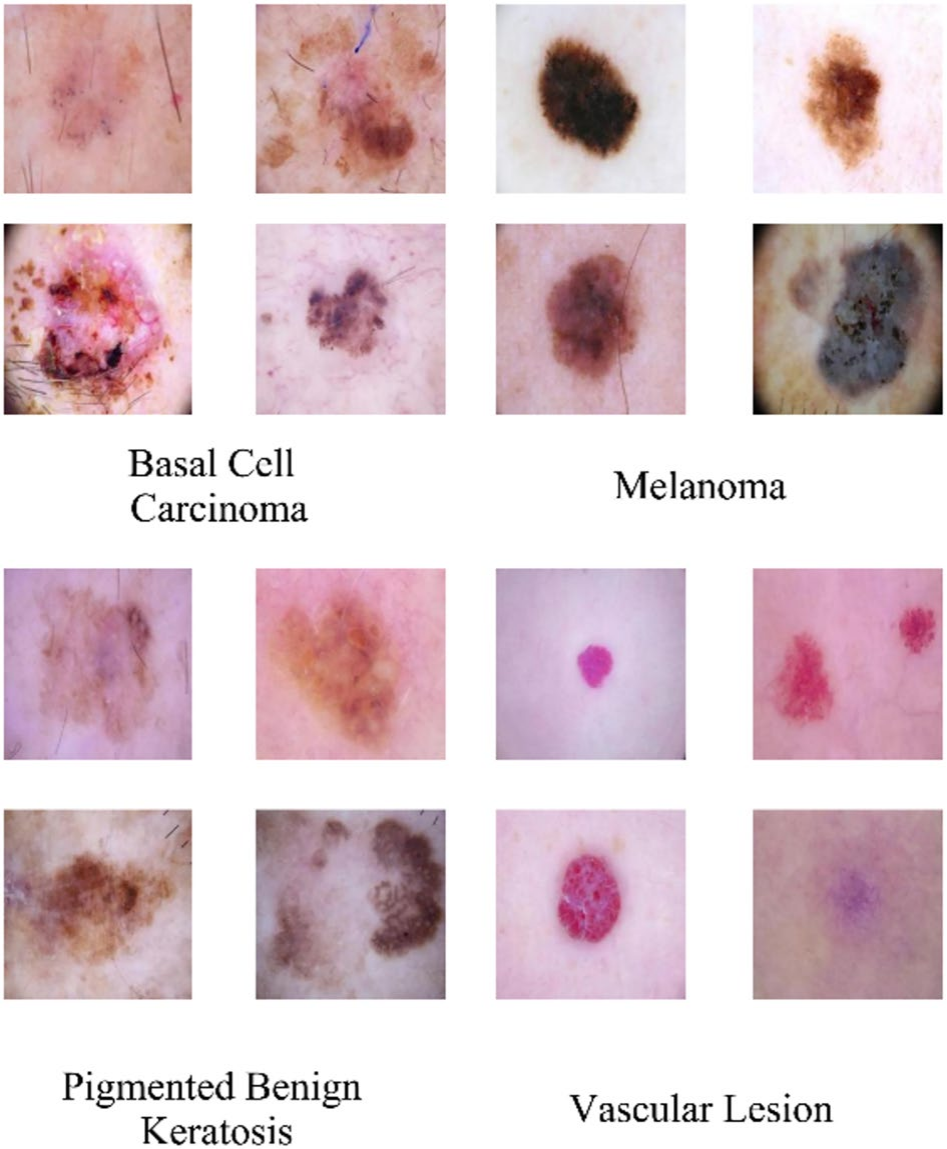
Examples of the investigated skin cancer classes: basal cell carcinoma, melanoma, pigmented benign keratosis, and vascular lesions according to the ISIC 2017.

The employed dataset was obtained from the esteemed International Skin Imaging Collaboration (ISIC), specifically from its 2017 release^57^. This rich dataset encompasses a diverse array of nine distinct classes, each representing a unique dermatological condition. The classes were actinic keratosis, basal cell carcinoma, dermatofibroma, melanoma, nevus, pigmented benign keratosis, seborrheic keratosis, squamous cell carcinoma, and vascular lesions. A total of 2357 images were meticulously distributed across these classes, creating a comprehensive repository of dermatoscopic visual data. In the process of feature extraction, certain classes are excluded due to their limited representation compared to the remaining classes. The refined subset of classes comprises basal cell carcinoma, melanoma, pigmented benign keratosis, and vascular lesions, amounting to a total of 1466 images. Figure 2 shows examples of the investigated skin cancer classes. To optimize the model's efficacy, the dataset was judiciously partitioned into distinct sets: 70% for training, 15% for validation, and 15% for testing purposes.

### Data processing

The initial phase in crafting the envisioned model involves managing the dataset by adjusting its dimensions to align seamlessly with the input size requirements of each employed deep learning model. Remarkably, the dataset was of commendable quality, rendering any augmentation or additional image processing enhancement strategies unnecessary. Its inherent excellence obviates the need for further refinement, affirming its suitability for direct integration into model development.

### Feature extraction for skin cancer images

A crucial choice about feature extraction from the input data must be made before a computer-aided diagnostic (CAD) system can be implemented. Two fundamental approaches have been adopted to address this challenge. The first approach revolves around crafting features manually, utilizing traditional, handcrafted techniques. This method, though meticulously executed, often results in a limited number of features that may not faithfully capture the intricacies of the input data. This approach, while precise, can sometimes fall short in representing the full spectrum of characteristics inherent to the data. The second involves harnessing the power of automated feature extraction through cutting-edge deep learning CNN algorithms. Leveraging CNNs unlocks the potential for generating an extensive array of features, offering a more comprehensive representation of each class or category within the dataset.

In our specific scenario, we have meticulously crafted and fine-tuned four distinct CNN models: AlexNet^23,40^, InceptionV3^9^, MobileNet V2^24,58^, and ResNet-50^2,43^. These models served as feature extractors for the representation of the various skin cancer classes under investigation. The quantity of extracted features is contingent upon the specific layer selected for feature extraction, effectively resulting in an output array denoted as $\left\{ {F_{1} F_{2} F_{3} \ldots \ldots . F_{N} } \right\}$. A comprehensive overview of the feature extraction phase is provided in Table 1. The table provides a summary of the four scenarios, encompassing the employed CNN model, the total count of extracted features, and the layer from which these features were derived. This approach enables us to precisely capture and differentiate the distinctive characteristics of the targeted skin cancer classes, enhancing the efficacy of the CAD system. In the feature extraction phase, the training parameters were configured for a comprehensive span of 30 epochs, employing an initial learning rate set at 0.0001 and following a piecewise learning rate schedule. Validation through a hands-on approach was conducted, and the weights were updated after every 600 iterations. Striking a balance between efficiency and accuracy, a judicious mini-batch size of 64 was selected for the optimization process.

**Table 1:** A summary of the feature extraction stages for skin cancer detection and classification.

Model	Number of features	Layer of extraction
AlexNet	4096	Fully connected no. 6
InceptionV3	2048	Average pooling
MobileNet V2	1280	Global average pooling
ResNet-50	2048	Global average pooling

### Feature selection for skin cancer images

The grey wolf optimizer (GWO)^49–51^ algorithm was employed to aid in feature extraction. By applying different deep learning models during the development of a CAD system, four distinct categories of skin cancer were identified. Inspired by the hunting behavior of grey wolves, the GWO algorithm was used to fine-tune the feature selection process, ensuring the selection of the most informative and discriminative features for the classification task. Several parameters govern the GWO algorithm, including population size, iteration count, exploration and exploitation rates, and convergence threshold^50^. These parameters play a pivotal role in guiding the search for an optimal subset of features, enhancing the performance of the CAD system in classifying skin cancer. These values are proposed based on the assumption that GWO2 involves a more aggressive reduction in the number of features than does GWO1. The convergence threshold has also been adjusted to reflect the potentially more challenging optimization problem in GWO2.

During this stage, a modification was performed to the classical GWO, particularly for coefficient ***a,*** as denoted by Eq. (23), to increase the exploration time for finding more solutions in the search space^51^. This algorithm was termed GWO2, while the algorithm related to the classical GWO was termed GWO1.

23 $$ a = 2{-}\left( {\frac{{2t^{2} }}{{T^{2} }}} \right) $$

**Figure 3:**
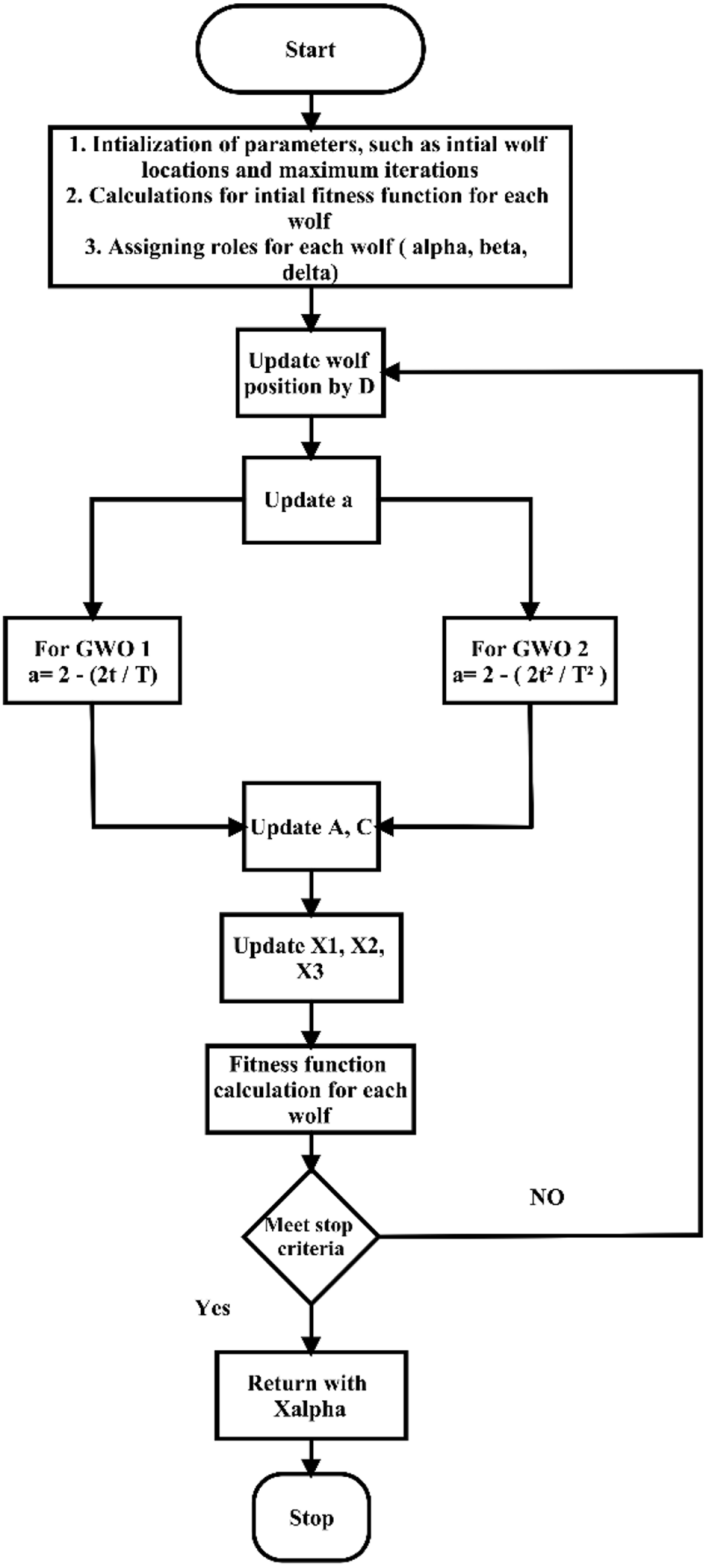
Flowchart of both versions of GWO for feature reduction in skin cancer images.

The two versions were employed to extract two feature maps for each deep learning model based on the control parameter settings, as presented in Table 2. As shown in Table 2, the exploration and exploitation rates, in addition to the convergence threshold, were altered between the two versions. The optimization process reduces the number of features retained as the most significant for the classification phase. According to the flow of the GWO, as shown in Fig. 3, the fitness function was calculated for each wolf for each iteration. The proposed fitness function for both algorithms is presented in Eq. (24).

24 $$ {\text{FGW}} = \frac{1}{{\text{Tmax }}}\mathop \sum \limits_{t = 1}^{{{\text{Tmax}}}} \left| {N \left( t \right)_{ref}^{label} - N^{label} \left( t \right)_{non } } \right| $$

where ***F*** is the fitness function, ***N***_***ref***_ is the output of the corresponding features for each labeled dermoscopic image, and ***N***_***non***_ is the output of the noncorresponding features for each labeled dermoscopic image.

**Table 2:** Controlling parameter settings for the two GWO algorithms.

Parameter	GWO 1 (classical)	GWO 2 (modified)
Population size	10	10
Number of iterations	100	100
Exploration rate	2	1.5
Exploitation rate	0.5	0.3
Convergence threshold	$1 \times e^{ - 6}$	$4 \times e^{ - 7}$

### Machine learning classification for skin cancer images

The fourth pivotal stage within the outlined framework entails the execution of image classification. This involves training an array of diverse ML classifiers, spanning from the linear SVM^59^ to the cubic SVM^43^ and further extending to the quadratic SVM^58^. Additionally, model development embraces the incorporation of a medium-sized neural network^60^, a wide neural network^61^, and an ensemble subspace discriminant^62^. This comprehensive approach reflects a nuanced strategy, leveraging an eclectic mix of classifiers to intricately capture and discern patterns within the dataset, fostering a robust and versatile classification model for CAD systems.

### Application of the ARAS method for selecting the optimum model

The final stage of the proposed methodology involves selecting the optimum model for image classification. According to the sequence of our methodology, we have four feature extractors and three feature maps: the original features and two maps due to the two versions of the GWO. All combinations of the feature extractor and feature map were applied to all the ML classifiers. As a result, 51 models were developed for the classification of skin cancer. In this way, we need to apply the ARAS method to select the optimum model. A detailed description of the ARAS method is given in Section “Multi-criteria decision-making approach”. According to the principles of the MCDM, a set of criteria was selected for model benchmarking. For this reason, nine evaluation metrics were chosen to distinguish the models. These included the accuracy (ACC), sensitivity (SV), specificity (SP), precision (PR), error rate (ER), false positive rate (FPR), false negative rate (FNR), negative predictive value (NPV), and F1 score (F1S)^9,59^. All criteria are identified in terms of true positives (TPs), false positives (FPs), true negatives (TNs), and false negatives (FNs), as shown in Eqs. (25)–(33).

25 $$ Accuracy = \left( {TP + TN} \right)/\left( {TP + FN + FP + TN} \right) $$

26 $$ Sensitivity = TP/\left( {TP + FN} \right) $$

27 $$ Errorrate = \left( {FP + FN} \right)/\left( {TP + FN + FP + TN} \right) $$

28 $$ Specificity = TN/\left( {TN + FP} \right) $$

29 $$ Precision = TP/\left( {TP + FP} \right) $$

30 $$ False\;Positive\;Rate = FP/\left( {FP + TN} \right) $$

31 $$ False\;Negative\;Rate = FN/\left( {FN + TP} \right) $$

32 $$ Negative\;Predictive\;Value = TN/\left( {TN + FN} \right) $$

33 $$ F1 - Score = \left( {2 \times \left( {Sensitivity \times Precision} \right)} \right)/\left( {Sensitivity + Precision} \right) $$

Obviously, criteria weighting is a pivotal step in implementing the ARAS method. According to previous studies, entropy and CRITIC are the dominant methods. Two attempts were made to use the CRITIC method in this study. The procedure of the CRITIC method is explained through the next steps^53^.

**Step 1**: Use the maximum and minimum values for each criterion, as indicated in Eq. (34), to normalize each ***x***_***ij***_ in the initial decision matrix to produce a normalized value ***r***_***ij***_.

34 $$ r_{ij} = \frac{{x_{ij} - x_{j}^{min} }}{{x_{j}^{max} - x_{j}^{min} }} $$

**Step 2**: A correlation coefficient is determined for all criteria as presented in (35). where ***σ*** is the standard deviation for each criterion ***j***.

35 $$ c_{j} = \sigma_{j} \mathop \sum \limits_{j = 1}^{m} \left( {1 - r_{ij} } \right) $$

**Step 3**: By normalizing the correlation coefficient, the criterion weight is computed as shown in Eq. (35).

36 $$ w_{j} = \frac{{c_{j} }}{{\mathop \sum \nolimits_{i = 1}^{m} c_{i} }} $$

### Declaration of generative AI and AI-assisted technologies in the writing process

During the preparation of this study, the authors partially used the QuillBot tool and a free trial of the American Journal Experts (AJE) (Springer Nature) platform for spelling and grammar checking. In addition, some sentences have been rephrased using these two services. After editing the text of the article, the authors reviewed and rewrote the contents of the article. The authors take full responsibility for the content of the article.

## Results and discussion

This study was conducted to classify four classes of skin cancer. A total of 1466 images related to the ISIC 2017 were used to classify basal cell carcinoma, melanoma, pigmented benign keratosis, and vascular lesions. A distinctive framework was developed by applying deep learning algorithms. Additionally, two versions of an optimization algorithm, six ML classifiers, and the RAPS method were applied. Three scenarios were adopted for feature extraction: the use of the original feature map, an optimized feature map due to the modified GWO called GWO2 and the classical optimizer known as GWO1. The numbers of selected features for each model for both algorithms are detailed in Table 3.

**Table 3:** Number of selected features for the two GWO algorithms compared to the original features.

Model	GWO1	GWO2	Original features
AlexNet	2248	1478	4096
InceptionV3	1476	683	2048
MobileNet V2	923	333	1280
ResNet-50	1420	776	2048

As noted in Table 3, for the AlexNet model, the number of features was reduced by 45% and 64% for GWO1 and GWO2, respectively. For the second model, the feature reductions were 28% and 67%, respectively. Considering the third model, the values were 28% and 74% for GWO1 and GWO2, respectively. Finally, for the ResNet-50 model, 31% and 64% of the samples were recorded for GWO1 and GWO2, respectively. These percentages revealed the performance of each algorithm separately. Furthermore, these findings clarify how strongly GWO2 affects feature selection. To this point, the modified GWO2 was faster even though it did not act in feature selection in the same way as the classical GWO1. On the other hand, the classical GWO1 algorithm performed better in feature selection, leading to an improvement in classification accuracy. However, the classical algorithm was more complex, making it more time-consuming to process. According to the hypothesis of the study, the classical GWO1 is the optimal one.

Within each of these scenarios, we meticulously trained six ML classifiers to regulate the classification process within the tailored CAD system. For AlexNet (A), Inception V3 (I), and ResNet 50 (R), we trained a cubic SVM (CSVM), wide neural network (WNN), quadratic SVM (QSVM), and medium neural network (MNN) separately for each scenario. Furthermore, for MobileNet V2 (M), we trained the CSVM, WNN, QSVM, linear SVM (LSVM), and ensemble subspace discriminant (ESD) models separately for each scenario. As a result, 51 models were developed for classifying skin cancer, as shown in Table 4. All the models were compared against the evaluation criteria. The computer used to conduct this study included an Intel Core i7, an NVIDIA GeForce MX 130, Windows 11, 64 bits, and 16 GB of RAM. Moreover, all the experiments were performed using the MATLAB R2021b program.

**Table 4:** Classification results of the developed models for skin cancer according to the three feature maps.

Fet. maps	No	Model	ACC	SV	SP	PR	ER	FPR	FNR	NPV	F1S
GWO1	1	A.CSVM	0.945	0.883	0.961	0.892	0.054	0.038	0.116	0.962	0.887
	2	A.WNN	0.945	0.899	0.961	0.908	0.054	0.039	0.100	0.961	0.903
	3	A.QSVM	0.942	0.877	0.958	0.897	0.057	0.041	0.122	0.959	0.885
	4	A.MNN	0.941	0.892	0.958	0.894	0.058	0.041	0.107	0.958	0.893
GWO2	5	A.CSVM	0.943	0.880	0.959	0.900	0.056	0.040	0.119	0.960	0.888
	6	A.WNN	0.941	0.887	0.958	0.898	0.058	0.042	0.112	0.958	0.892
	7	A.QSVM	0.938	0.872	0.956	0.890	0.061	0.044	0.127	0.956	0.879
	8	A.MNN	0.939	0.878	0.956	0.897	0.060	0.043	0.122	0.957	0.887
Orig.	9	A.CSVM	0.945	0.883	0.960	0.903	0.054	0.039	0.116	0.961	0.892
	10	A.WNN	0.943	0.892	0.959	0.900	0.057	0.040	0.108	0.959	0.895
	11	A.QSVM	0.940	0.875	0.957	0.893	0.059	0.042	0.124	0.958	0.883
	12	A.MNN	0.940	0.885	0.957	0.895	0.059	0.042	0.114	0.957	0.890
GWO1	13	I. CSVM	0.925	0.826	0.947	0.855	0.074	0.052	0.173	0.949	0.837
	14	I.WNN	0.921	0.834	0.945	0.841	0.078	0.055	0.165	0.945	0.837
	15	I.QSVM	0.920	0.821	0.943	0.840	0.079	0.056	0.178	0.944	0.829
	16	I.MNN	0.920	0.838	0.944	0.832	0.079	0.055	0.161	0.944	0.835
GWO2	17	I. CSVM	0.915	0.809	0.940	0.836	0.084	0.060	0.190	0.941	0.820
	18	I.WNN	0.916	0.826	0.941	0.825	0.083	0.058	0.173	0.941	0.826
	19	I.QSVM	0.913	0.806	0.938	0.835	0.086	0.061	0.193	0.939	0.817
	20	I.MNN	0.884	0.703	0.914	0.702	0.115	0.085	0.296	0.914	0.702
Orig.	21	I. CSVM	0.929	0.840	0.950	0.862	0.070	0.049	0.159	0.951	0.850
	22	I.WNN	0.928	0.846	0.950	0.852	0.071	0.050	0.153	0.950	0.849
	23	I.QSVM	0.929	0.846	0.950	0.861	0.070	0.049	0.153	0.951	0.852
	24	I.MNN	0.925	0.841	0.948	0.847	0.074	0.052	0.158	0.948	0.844
GWO1	25	M.CSVM	0.885	0.764	0.917	0.804	0.115	0.082	0.235	0.919	0.782
	26	M.WNN	0.886	0.776	0.919	0.781	0.114	0.080	0.223	0.919	0.778
	27	M.QSVM	0.883	0.764	0.916	0.795	0.116	0.083	0.235	0.917	0.778
	28	M.LSVM	0.886	0.765	0.918	0.802	0.114	0.081	0.235	0.919	0.781
	29	M.SD	0.861	0.727	0.902	0.733	0.138	0.097	0.272	0.902	0.730
GWO2	30	M.CSVM	0.869	0.730	0.907	0.765	0.130	0.093	0.269	0.908	0.745
	31	M.WNN	0.864	0.735	0.904	0.740	0.136	0.095	0.264	0.904	0.737
	32	M.QSVM	0.869	0.732	0.907	0.762	0.130	0.092	0.267	0.908	0.745
	33	M.LSVM	0.874	0.732	0.910	0.787	0.125	0.090	0.267	0.911	0.755
	34	M.SD	0.869	0.748	0.907	0.760	0.130	0.092	0.251	0.908	0.754
Orig.	35	M.CSVM	0.882	0.760	0.915	0.799	0.117	0.084	0.239	0.917	0.777
	36	M.WNN	0.882	0.774	0.916	0.779	0.117	0.083	0.225	0.916	0.776
	37	M.QSVM	0.882	0.762	0.916	0.795	0.117	0.084	0.237	0.916	0.777
	38	M.LSVM	0.885	0.759	0.918	0.801	0.114	0.081	0.240	0.919	0.777
	39	M.SD	0.834	0.686	0.883	0.672	0.165	0.116	0.313	0.883	0.678
GWO1	40	R. CSVM	0.929	0.844	0.949	0.864	0.070	0.050	0.155	0.951	0.851
	41	R.WNN	0.931	0.850	0.950	0.870	0.068	0.049	0.149	0.952	0.858
	42	R.QSVM	0.932	0.863	0.952	0.858	0.067	0.047	0.136	0.952	0.861
	43	R.MNN	0.943	0.874	0.960	0.881	0.056	0.039	0.125	0.960	0.875
GWO2	44	R. CSVM	0.928	0.850	0.949	0.862	0.071	0.050	0.150	0.950	0.854
	45	R.WNN	0.924	0.835	0.945	0.856	0.075	0.054	0.164	0.947	0.842
	46	R.QSVM	0.927	0.846	0.948	0.859	0.072	0.051	0.154	0.949	0.850
	47	R.MNN	0.926	0.847	0.948	0.851	0.073	0.051	0.152	0.948	0.849
Orig.	48	R. CSVM	0.932	0.854	0.951	0.869	0.067	0.048	0.145	0.952	0.859
	49	R.WNN	0.932	0.852	0.951	0.869	0.067	0.048	0.147	0.952	0.858
	50	R.QSVM	0.932	0.856	0.951	0.872	0.067	0.048	0.143	0.952	0.862
	51	R.MNN	0.9308	0.8578	0.9513	0.8568	0.0692	0.0487	0.1422	0.9514	0.8570

Due to the many models, the RAPS method was implemented along with the CRITIC method to identify the superior model for classifying skin cancer images. Initially, beneficial and nonbeneficial criteria should be addressed before RAPS implementation. The accuracy, sensitivity, specificity, precision, and F1 score are beneficial criteria, while the error rate, false positive rate, false negative rate, and negative predictive value are nonbeneficial criteria. Criteria weights were calculated by the CRITIC method using Eqs. (33)–(35), as shown in Table 5.

**Table 5:** Criteria weights determined using the CRITIC method for selecting the optimum model for skin cancer classification.

	ACC	SV	SP	PR	ER	FPR	FNR	NPV	F1S
Correlation coefficient	1.321	1.427	1.349	1.203	2.672	2.719	2.901	1.579	1.512
Weight	0.079	0.086	0.081	0.072	0.160	0.163	0.174	0.095	0.091

**Figure 4:**
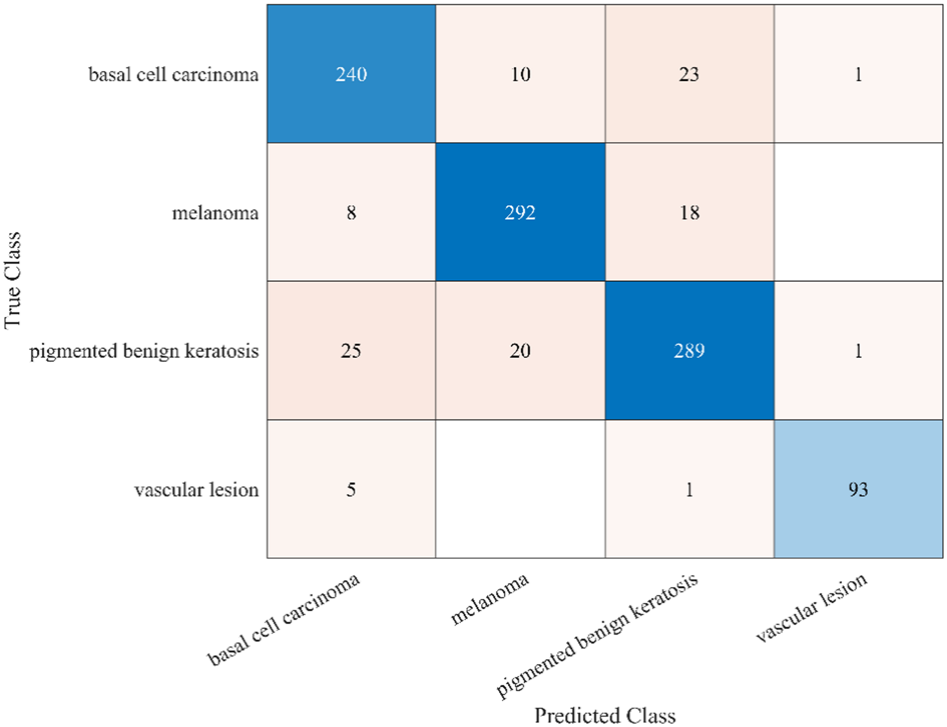
The confusion matrix of the best model (AlexNet-GWO1-WNN) of classifying skin cancer images.

**Figure 5:**
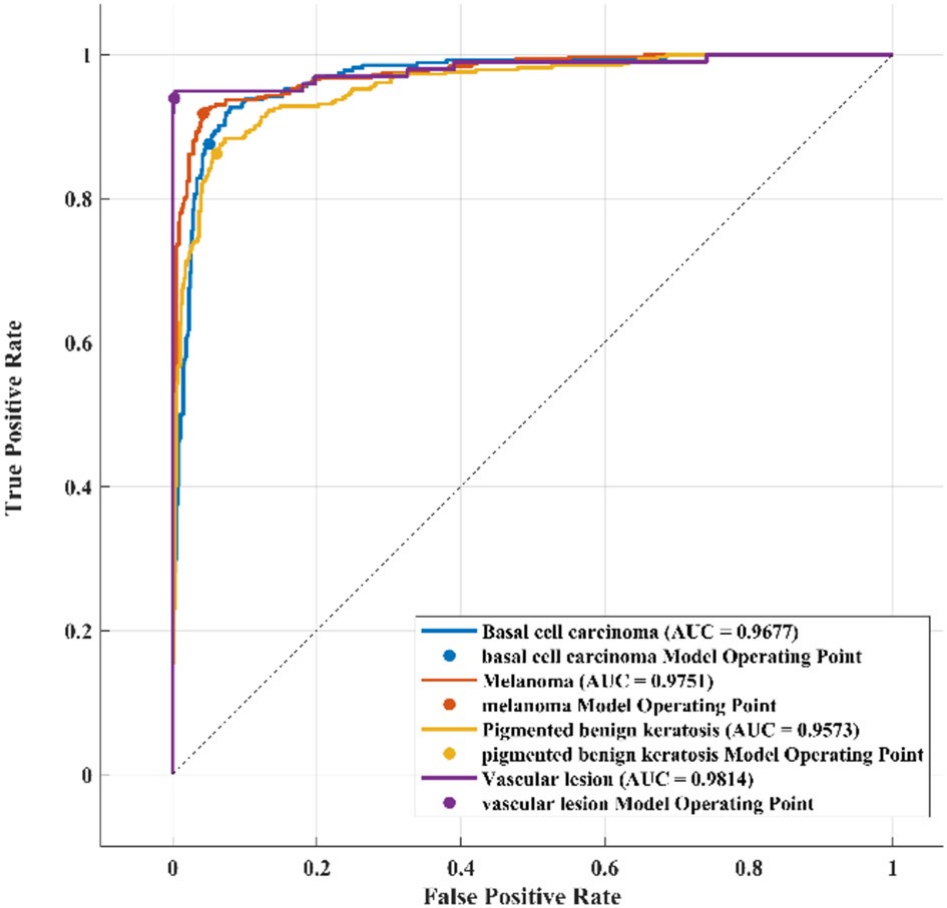
The ROC and AUC of the best model (AlexNet-GWO1-WNN) of classifying skin cancer images.

To demonstrate the application of the RAPS method, Table 6 presents samples of method implementation through step 5, considering only the best and worst cases. Taking this into account, step 4 was executed according to Eq. (14) to produce 0.18316 and 0.3023 for Qk (max) and Qh (min), respectively. Using Eq. (20), the *P* value equals 0.8389. Steps 6 and 7 are presented in Table 7, where “A2” represents Model 2 and “A34” represents Model 34. The final rankings of the 51 models are listed in Table 8. As a result, the confusion matrix of the best model, the ROC, and the AUC are presented in Figs. 4 and 5, respectively.

**Table 6:** An example of implementing the RAPS method for skin cancer classification for models 2 and 34.

	Step	ACC	SV	SP	PR	ER	FPR	FNR	NPV	F1S
Weight		0.079	0.086	0.081	0.072	0.160	0.163	0.174	0.095	0.091
Max/Min		0.945	0.899	0.962	0.908	0.038	0.038	0.100	0.883	0.903
A2		0.945	0.899	0.961	0.908	0.054	0.039	0.100	0.961	0.903
A34		0.869	0.748	0.907	0.760	0.130	0.092	0.251	0.908	0.754
A2	Step 1	0.999	1.000	0.999	1.000	0.704	0.987	1.000	0.919	1.000
A34		0.919	0.832	0.944	0.837	0.295	0.417	0.400	0.973	0.834
A2	Step 2	0.079	0.085	0.081	0.072	0.112	0.160	0.173	0.087	0.090
A34		0.072	0.071	0.076	0.060	0.047	0.067	0.069	0.092	0.075
Qmax	Step 3	0.079	0.086	0.081	0.072					0.091
Qmin						0.160	0.163	0.174	0.095	
A2Umax	Step 5	0.079	0.085	0.080	0.072					0.090
A2Umin						0.112	0.160	0.173	0.087	
A34Umax		0.072	0.071	0.076	0.060					0.834
A34Umin						0.047	0.067	0.069	0.092

**Table 7:** Ranking of the skin cancer classifications for models 2 and 34 based on the RAPS method.

Model	Qk(i)max	Qh(i)max	Pi	PSi	Rank
A2Umax	0.18313	0	0.79121	0.94313	1
A2Umin	0	0.27646	0		
A34Umax	0.07565	0	0.37880	0.45154	51
A34Umin	0	0.14213	0

**Table 8:** Final rankings of all the developed models for skin cancer classification based on the RAPS method.

Model no	Model	PSi	Rank
**A2**	**GWO1- AlexNet – WNN**	**0.943135**	**1**
A10	Original-AlexNet – WNN	0.914284	2
A1	GWO1-AlexNet – CSVM	0.911944	3
A9	Original -AlexNet – CSVM	0.909893	4
A35	Original -Mobile V2 – CSVM	0.907412	5
A4	GWO 1- AlexNet – MNN	0.907079	6
A6	GWO 2- AlexNet – WNN	0.893249	7
A5	GWO 2- AlexNet – CSVM	0.893027	8
A43	GWO 1- ResNet 50 – QSVM	0.886682	9
A12	Original- AlexNet – MNN	0.885985	10
A3	GWO 1- AlexNet – QSVM	0.881655	11
A11	Original- AlexNet – QSVM	0.86874	12
A8	GWO 2- AlexNet-MNN	0.867611	13
A7	GWO 2- AlexNet – QSVM	0.854574	14
A42	GWO 1- ResNet 50 – MNN	0.81751	15
A50	Original- ResNet 50 – QSVM	0.809088	16
A51	Original- ResNet 50 – WNN	0.804455	17
A48	Original-ResNet 50 – CSVM	0.803863	18
A49	Original -ResNet 50 – LSVM	0.801991	19
A41	GWO 1- ResNet 50 – LSVM	0.79677	20
A23	Original- Inception V3 – QSVM	0.788236	21
A44	GWO 2- ResNet 50 – CSVM	0.78679	22
A40	GWO 1- ResNet 50 – CSVM	0.784624	23
A22	Original – Inception V3 – WNN	0.784136	24
A21	Original – Inception V3 – CSVM	0.782796	25
A47	GWO 2 – ResNet 50 – WNN	0.777952	26
A46	GWO 2 – ResNet 50 – QSVM	0.777777	27
A24	Original – Inception V3 – MNN	0.770182	28
A45	GWO 2 – ResNet 50 – LSVM	0.758805	29
A13	GWO 1 – Inception V3 – CSVM	0.756305	30
A16	GWO 1 – Inception V3 – MNN	0.751902	31
A14	GWO 1 – Inception V3 – WNN	0.751277	32
A15	GWO 1 – Inception V3 – QSVM	0.73575	33
A18	GWO 2 – Inception V3 – WNN	0.731325	34
A17	GWO 2 – Inception V3 – CSVM	0.715933	35
A19	GWO 2 – Inception V3 – QSVM	0.708347	36
A26	GWO 1 – Mobile V2 – WNN	0.645514	37
A28	GWO1 – MobileNet V2 – LSVM	0.640933	38
A36	Original – MobileNet V2 – WNN	0.640323	39
A25	GWO1 – MobileNet V2 – CSVM	0.63993	40
A38	Original – MobileNet V2 – LSVM	0.637589	41
A27	GWO 1 – MobileNet V2 – QSVM	0.637512	42
A37	Original – MobileNet V2 – QSVM	0.63563	43
A33	GWO 2- MobileNet V2 – LSVM	0.614532	44
A30	GWO 2- MobileNet V2 – CSVM	0.607877	45
A32	GWO 2 – MobileNet V2 – QSVM	0.607588	46
A31	GWO 2 – MobileNet V2 – WNN	0.603945	47
A20	GWO 2 – InceptionNet V3 – MNN	0.603502	48
A29	GWO 1 – MobileNet V2 – ESD	0.598146	49
A39	Original – MobileNet V2 – ESD	0.564224	50
A34	GWO 2 – MobileNet V2 – ESD	0.45154	51

As indicated in Table 6, the best model comprised GWO1 with AlexNet and a wide neural network, and the worst model was GWO2 with MobileNet V2 and an ensemble subspace discriminant. Although the results differ among the grey wolf optimizers, feature extractors, and classifiers, GWO 1, AlexNet, and the wide neural network achieved outstanding performances, with an accuracy of 94.5%, a sensitivity of 90%, and a specificity of 96%, as depicted in Table 4.

**Figure 6:**
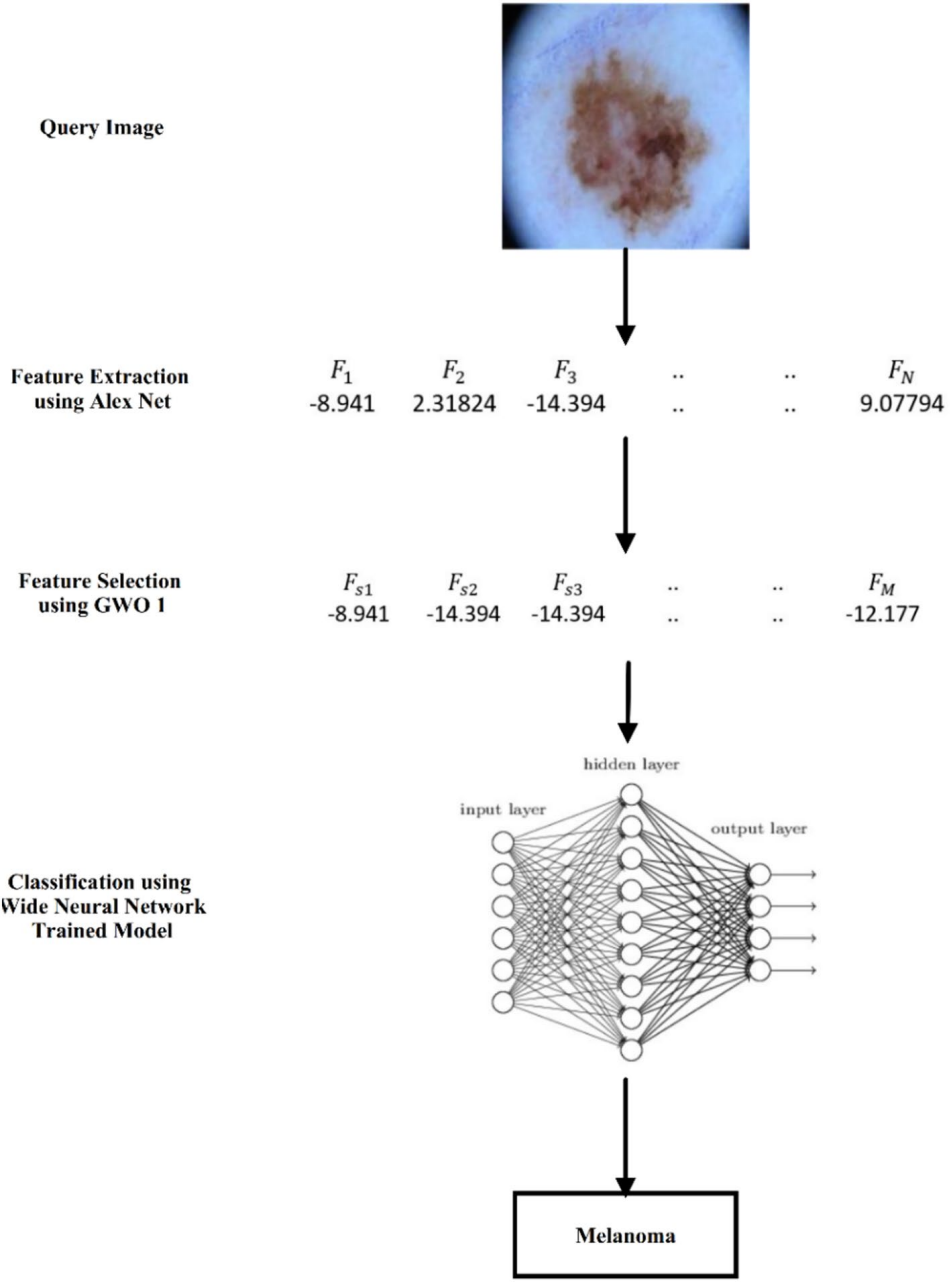
Overall summary of skin cancer classification using the proposed methodology.

This can be interpreted as follows: compared to the complex architectures of its successors, the structure of AlexNet is relatively straightforward. This transparency allows easier interpretation of the extracted features, providing valuable insights into the model's decision-making process. The utilized dataset is limited in size compared to other image recognition tasks. AlexNet’s ability to perform well with smaller datasets makes it a suitable choice for medical applications where data availability might be a constraint. In the challenge of accurate skin cancer classification, WNNs have yielded promising results, outperforming other models, such as LSVM, QSVM, CSVM, and ESD. While SVMs offer interpretability and robustness, they struggle with complex, nonlinear relationships often present in skin lesions. ESD tackles high dimensionality well but is limited by interpretability and cost. WNNs, however, shine with their ability to capture these intricate patterns, boasting greater accuracy thanks to their massive learning capacity and end-to-end learning. While challenges such as overfitting and interpretability linger, the sheer power and potential of WNNs make them the dominant force in this crucial medical application. The outcome results based on the proposed methodology are shown in Fig. 6. The proposed methodology indicated that the AlexNet, GWO1, and WNN combination was the most effective. Consequently, Fig. 6 provides an example of an application, showcasing the feature reduction and identifying the class of the image under test. For feature reduction, for instance, the original feature map was 4096 and after applying the GWO1 became 2248.

When comparing our study's findings to those of related studies, numerous disparities emerge. Almost all the studies were differentiated according to the network utilized. In^18^, the inception and residual networks were used; however, in^3^, the authors modified Inception-ResNet V1 to detect skin cancer. Another network, namely, the stacked CNN, was used to detect melanoma classes^5^. A novel CNN based on VGG16 was developed to detect melanoma^29^. The same was conducted in^31^ for developing a novel CNN for skin cancer classification. According to our proposed methodology, we used four CNN algorithms for feature selection and six ML algorithms for skin cancer classification. Therefore, the difference lies not only in the utilized algorithms but also in the modeling sequences. Additionally, we employed two versions of GWO to optimize feature selection, reduce the training time, and improve the classification process. The optimization context was adopted in^27^ using the whale optimization algorithm, and the artificial rabbit optimizer was also used for another relevant work^4^. Despite the investigation of cancer classification problem with various models, such as in^63–65^, the MCDM approach has not been used to select the best model, in particular skin cancer classification. According to the proposed methodology, 51 models were found to represent the skin cancer classification. A recent method called RAPS was implemented to select the superior model. This study is the first to present a skin cancer classification model among 51 models, leading to the use of the MCDM to select a superior model. Unlike backpropagation optimization techniques^66–68^, swarm intelligence-based optimization techniques have been adopted, and they proved their robustness in feature selection.

## Conclusions

This study's conclusion involved the classification of skin cancer based on a variety of methods. The authors applied deep learning, machine learning, an optimization algorithm, and the MCDM to develop multiple models and select the best model. AlexNet, Inception V3, MobileNet V2, and ResNet 50 were employed as feature extractors. For feature selection, three scenarios were used: two with the GWO and one without an optimizer. Six different ML classifiers were used to classify skin cancer samples as basal cell carcinoma, melanoma, pigmented benign keratosis, or vascular lesions. Therefore, many models have been developed because of their distinct arrangements. A recent method called the RAPS was applied to select the optimum model for skin cancer classification. The arrangement of GWO1 with AlexNet and WNN yielded the best results. The proposed framework was tested on the ISIC 2017 dataset. The findings validate the applicability of the suggested framework. The development of multiple models for disease diagnosis provides a wide spectrum of models for precisely selecting the fittest model. Furthermore, feature reduction plays an influential role in the rapid detection and/or classification of skin cancer images. Even though feature reduction shortens training times, it does not necessarily guarantee the best performance of the developed models. Additionally, developing CAD systems can assist dermatologists in properly differentiating various classes of skin cancer, which in turn reduces errors and aids in providing correct treatment protocols. Future work can use other settings for feature extraction and selection, in addition to validation with other datasets for different or the same skin cancer classes. Moreover, other optimization types and techniques could be considered to identify their impacts on feature selection. Additionally, other recent MCDM methods can be investigated for model selection. Model deployment via web-based services can be implemented to enhance disease diagnosis by facilitating more convenient tools, such as the applications of telemedicine. This study can be extended to other types of cancer or other diseases.

## Data Availability

The datasets analyzed during the current study are available in the ISIC 2017 repository, 10.1109/ISBI.2018.8363547.

## Abbreviations

ABC: Artificial Bee Colony
ACO: Ant Colony Optimization
AHP: Analytic Hierarchy Process
AUC: Area Under the Curve
CAD: Computer-Aided Diagnosis
CBR: Case-Based Reasoning
CNN: Convolutional Neural Networks
CRITIC: Criterion Importance via Intercriteria Correlation
CSVM: Cubic Support Vector Machine
DEA: Data Envelopment Analysis
DOLHGS: Dynamic Opposite Learning Hunger Games Search
ELECTRE: Elimination and Choice in Expressing Reality
ESD: Ensemble Subspace Discriminant
FC: Fully Connected
FFO: Fruit Fly Optimization
GWO: Grey Wolf Optimizer
HGS: Hunger Games Search
HHO: Harris Hawk Optimizer
IOU: Intersection Over Union
ISIC: International Skin Imaging Collaboration
K-NN: K-Nearest Neighbor
LSVM: Linear Support Vector Machine
MCDM: Multi Criteria Decision Making
ML: Machine Learning
MNN: Medium Neural Network
MOORA: Mult Objective Optimization by Ratio Analysis
PROMETHEE: Preference Ranking Organization Method for Enrichment Evaluation
QSVM: Quadratic Support Vector Machine
RAPS: Ranking the Alternatives by Perimeter Similarity
ROC: Receiver Operating Characteristic
SAW: Simple Additive weighting
SVM: Support Vector Machine
TOPSIS: Technique for Order Preference by Similarity to the Ideal Solution
WNN: Wide Neural Network
WOA: Whale Optimization Algorithm
